# Th1 cells reduce the osteoblast-like phenotype in valvular interstitial cells by inhibiting NLRP3 inflammasome activation in macrophages

**DOI:** 10.1186/s10020-024-00882-z

**Published:** 2024-07-30

**Authors:** Jing Lu, Jiaming meng, Gang Wu, Wulong Wei, Huabao Xie, Yanli Liu

**Affiliations:** 1 The First Clinical Medical College, Guangxi Medical University, Guangxi Zhuang Autonomous Region, Shuangyong Road 22, Nanning, 530021 P.R. China; 2 Department of Cardiology, Liuzhou People’s Hospital, Guangxi, Zhuang Autonomous Region, Wenchang Road 8, Liuzhou, 545000 P.R. China

**Keywords:** Th1, IFN-γ, Calcific aortic valve stenosis, NLRP3, Macrophages

## Abstract

**Background and aims:**

Inflammation is initiates the propagation phase of aortic valve calcification. The activation of NLRP3 signaling in macrophages plays a crucial role in the progression of calcific aortic valve stenosis (CAVS). IFN-γ regulates NLRP3 activity in macrophages. This study aimed to explore the mechanism of IFN-γ regulation and its impact on CAVS progression and valve interstitial cell transdifferentiation.

**Methods and results:**

The number of Th1 cells and the expression of IFN-γ and STAT1 in the aortic valve, spleen and peripheral blood increased significantly as CAVS progressed. To explore the mechanisms underlying the roles of Th1 cells and IFN-γ, we treated CAVS mice with IFN-γ-AAV9 or an anti-IFN-γ neutralizing antibody. While IFN-γ promoted aortic valve calcification and dysfunction, it significantly decreased NLRP3 signaling in splenic macrophages and Ly6C^+^ monocytes. In vitro coculture showed that Th1 cells inhibited NLPR3 activation in ox-LDL-treated macrophages through the IFN-γR1/IFN-γR2-STAT1 pathway. Compared with untreated medium, conditioned medium from Th1-treated bone marrow–derived macrophages reduced the osteogenic calcification of valvular interstitial cells.

**Conclusion:**

Inhibition of the NLRP3 inflammasome by Th1 cells protects against valvular interstitial cell calcification as a negative feedback mechanism of adaptive immunity toward innate immunity. This study provides a precision medicine strategy for CAVS based on the targeting of anti-inflammatory mechanisms.

**Graphical Abstract:**

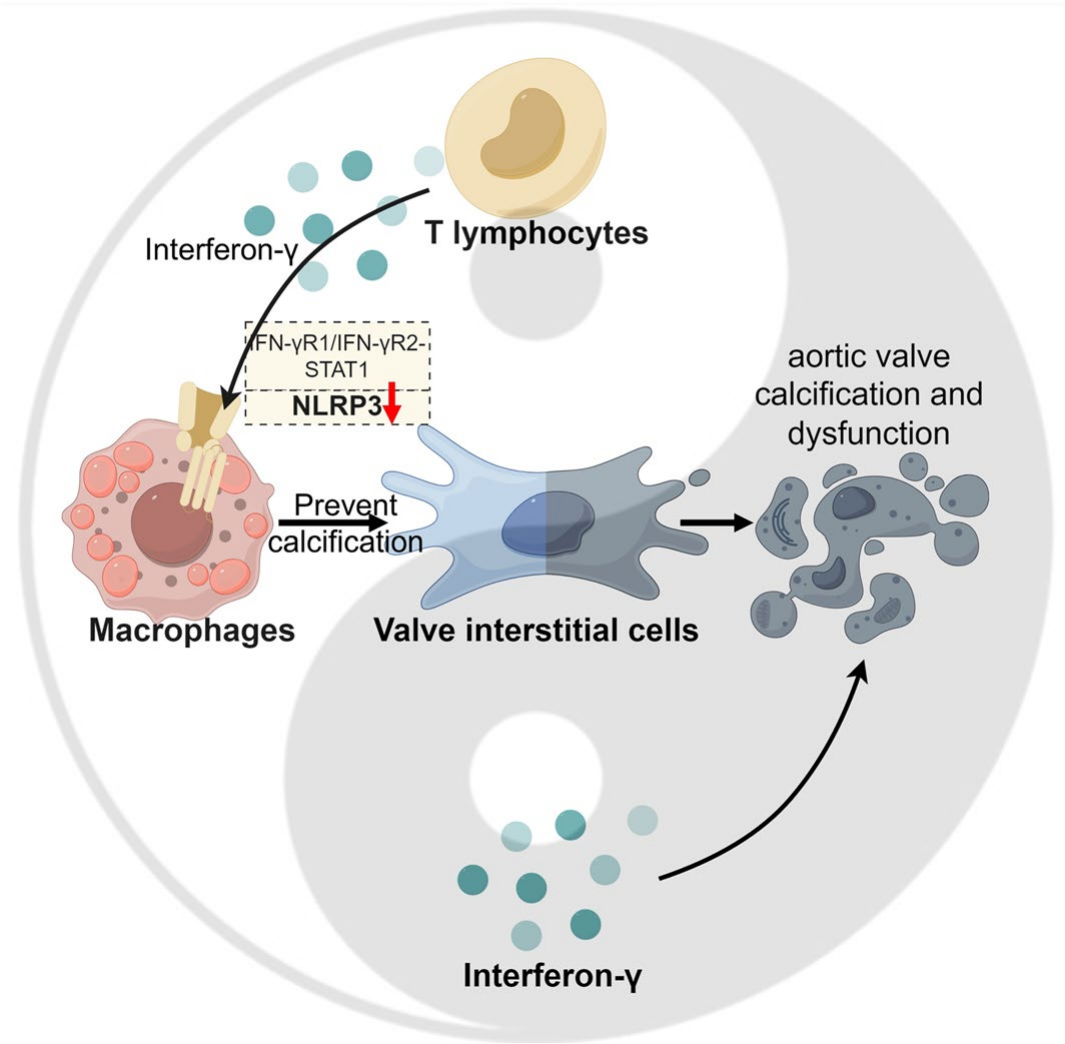

**Supplementary Information:**

The online version contains supplementary material available at 10.1186/s10020-024-00882-z.

## Introduction

Calcific aortic valve stenosis (CAVS) is the most common heart valve disease in elderly individuals. It is mainly characterized by aortic valve leaf fibrosis, calcification and the accumulation of osteogenic nodules (Wasilewski et al. 2012). In developed countries, as many as 25% of those over 65 years of age have CAVS, and 2–5% of patients with severe aortic stenosis need surgical treatment (Taniguchi et al. 2015). Due to the lack of early prevention strategies and drug treatments in the clinic, aortic valve replacement can only be performed once symptoms of cardiac decompensation appear. The implanted valve has a limited service life after surgery, and long-term anticoagulant use is required (Walther et al. 2012).

The pathological mechanism of CAVS is related to age, mechanical pressure, lipid deposition and early calcium regulation (Miller et al. 2011). Immunity plays an important role in the initiation and progression of the pathological process of calcification (Goody et al. 2020). Cytokines secreted by macrophages can regulate the differentiation of valve interstitial cells into myofibroblast-like or osteoblast-like cells (Li et al. 2017; Grim et al. 2020). In addition, the proportion of intermediate CD14^+^CD16^+^ monocytes increase in patients with severe aortic stenosis, which is associated with worse cardiac function, and decreases significantly after transcatheter aortic valve replacement(Hewing et al. 2017a, b). Single-cell RNA sequencing revealed that the gene expression of the NLRP3 inflammasome in peripheral blood monocytes is increased in patients with severe CAVS(Abplanalp et al. 2020). NLRP3, which is mainly expressed on monocytes and macrophages, is an important part of the innate immune response and participates in the initiation and activation of inflammatory signals (Ting et al. 2008). The NLRP3 inflammasome, as a pattern recognition receptor in the cytoplasm, recognizes pathogen-associated molecular patterns (PAMPs) and damage-associated molecular patterns (DAMPs) and recruits and activates the protease caspase-1 by binding to the adaptor protein apoptosis-associated speck-like protein containing a CARD (ASC) through the N-terminal pyrin domain. Activated caspase-1 cleaves the precursors of IL-1β and IL-18 to produce the corresponding mature cytokines. The NLRP3 inflammasome is related to the occurrence and pathogenesis of infectious and chronic degenerative diseases (Swanson et al. 2019). We previously reported that blocking the NLRP3 inflammasome inhibited M1 macrophage polarization and reduced osteogenic calcification, indicating that NLRP3 inflammasome activation in macrophages facilitates CAVS progression (Lu et al. 2022).

In the adaptive immune response, IFN-γ is the main effector cytokine that performs the function of CD4^+^ T helper type 1 (Th1) cells. IFN-γ and LPS stimulate macrophages to produce proinflammatory M1 subsets in vitro, mimicking the activation of monocytes and macrophages to a proinflammatory state by IFN-γ. IFN-γ, a proinflammatory cytokine in vascular disease, not only promotes AS but also exacerbates valve calcification in CAVS through the JAK/STAT signaling pathway and hypoxia-inducible factor-1α pathway in synergy with TLR3 and TLR4 agonists (Parra-Izquierdo et al. 2019, 2021; Zhou et al. 2015). However, other studies have shown that myeloid IFN-γ receptor 2 (IFN-γR2) deficiency does not affect atherosclerosis in HFD-fed mice (Boshuizen et al. 2016). Recent studies have reported that transcriptional heterogeneity and immune cell function contribute to the dual roles of IFN-γ. In atherosclerosis, IFN-γ induces proinflammatory M(IFN-γ)i subsets with pathogenic effects and phagocytosis-characterized M(IFN-γ)p subsets with alleviating effects (Decano et al. 2023). Precise targeting of IFN-γ to different heterogeneous subpopulations or different signaling pathways in monocytes and macrophages produces different results. IFN-γ can counteract IL-1β signaling at multiple levels: IL-1β mRNA transcription, NLRP3 inflammasome activation, and IL-1R antagonism (Labzin et al. 2016). Eigenbrod et al. reported that IFN-γ can block IL-1β mRNA transcription by inhibiting the IL-1β promoter (Eigenbrod et al. 2013). IFN-γ can not only inhibit the transcription of IL-1β precursors but can also specifically inhibit the activation of the NLRP3 inflammasome dependent on nitric oxide (NO), thereby inhibiting the maturation of IL-1β (Mishra et al. 2013). The inhibitory effect of IFN-γ on the NLRP3 inflammasome is an important negative feedback mechanism for the body’s inflammatory response after the initiation of the adaptive immune response. Other research has shown that death-associated protein kinase 1 inhibits the activation of NLRP3 by activating IFN-γ to restrain inflammatory pathology during fungal infection (Oikonomou et al. 2016). The above results suggest that there is a connection between IFN-γ and the NLRP3 inflammasome in macrophages.

In this study, we detected the frequency of Th1 cells and changes in the expression of IFN-γ and STAT1 in the aortic valve. To investigate the correlation between IFN-γ expression levels and macrophage NLRP3 inflammasome activation, we used adenovirus to overexpress IFN-γ and neutralizing antibodies to reduce IFN-γ levels. In vitro, Th1 cells were cocultured with ox-LDL-induced bone marrow–derived macrophages (BMDMs) to observe the activation of NLRP3 in BMDMs and the effect of secretory mediators on the transdifferentiation of valve interstitial cells.

## Method

### Animal model

Six-week-old male ApoE^−/−^ mice and wild-type (WT) mice with a C57BL/6J background (identification number: SCXK2016-0006) were procured from Vital River Laboratory Animal Technology (Beijing, China). These mice were housed under specific pathogen-free conditions at the Guangxi Medical University Laboratory Animal Centre. The experiments were conducted in strict accordance with the Experimental Animal Ethics guidelines of Guangxi Medical University. The experimental protocols strictly adhered to the ARRIVE guidelines for transparent and comprehensive reporting of animal research. ApoE^−/−^ mice were divided into CAVS groups and fed a high-fat diet (HFD) for 12, 24 and 36 weeks, respectively; the HFD was obtained from Jiangsu Xietong Pharmaceutical Bioengineering Co., Ltd., Nanjing, China, as described previously. The WT mice in the control group were fed a standard normal diet.

### Adeno-associated virus 9 (AAV9) for in vivo administration and neutralizing antibodies against IFN-γ

To overexpress IFN-γ, recombinant AAV9 expressing IFN-γ and AAV9-GFP were purchased from Shanghai Jikai Co. (Shanghai, China). The titers of the AAV vectors (viral genomes/ml) were determined by quantitative real-time PCR. AAV9-NC, which was packed with empty AAV9 virus, was used as a negative control. Mice were injected with 2 × 10^13^ VG of AAV9- IFN-γ or AAV9-NC twice: at weeks 0 and 12 of the HFD(Knezevic et al. 2016). The hearts were harvested from all injected mice 3 weeks after the second injection to examine IFN-γ expression by western blotting or immunofluorescence staining. For neutralizing antibody intervention, mice were injected with received the 100 µg of the anti-IFN-γ antibody (Cat. BE0055; BioXcell, USA) intraperitoneally (i.p.) every 3 days from week 0 to week 24 of the HFD. Vehicle mice were injected with InVivoPure pH 7.0 Dilution Buffer (Cat. IP0070; BioXcell, USA) which was used to dilute the solvent. All mice treated with AAV9 or neutralizing IFN-γ antibodies were killed at week 24, and the hearts, spleens and peripheral blood were collected for further experiments.

### Echocardiography

Aortic valve function was assessed with a MyLab™ Sat ultrasonic imaging system equipped with a 22-MHz probe (Esaote, Genova, Italy). Cardiac echocardiography was performed at 12, 24, and 36 weeks. During cardiac echocardiography, the mice underwent mild anesthesia through the i.p. injection of 10 µl of 1.25% avertin solution per gram of body weight. The heart rate was maintained at 490 ± 30 beats/minute. Aortic valve flow velocity was assessed by continuous-wave Doppler recorded from a five-chamber view via the apical approach and averaged over 5 beats. M-mode echocardiograms were employed to capture aortic valve systolic dimensions and left ventricular function. The aortic valve shape was observed on transverse sections of the heart, and the mean aortic valve area was recorded and calculated. The data were collected and analyzed by a skilled sonographer following the double-blind principle. To determine the left ventricular end-diastolic volume (LVEDV) and end-systolic volume (LVESV), the Teichholz formula was used.

### Histology

To evaluate calcium deposits and collagen deposition in mouse aortic valve tissue, paraffin-embedded hearts were sectioned into 5-µm-thick slices comprising three valve leaflets. These sections were stained with hematoxylin and eosin (HE), von Kossa (Solarbio, Beijing, China), and Masson (Solarbio, Beijing, China) following the manufacturer’s guidelines. Quantitative analysis of the staining was performed using Image-Pro Plus software (Media Cybernetics, Bethesda, USA) to determine the percentage of von Kossa-positive and Masson-positive staining areas.

### Immunohistochemistry (IHC)

Tissue sections of the aortic valve were subjected to heat-induced epitope retrieval and then incubated overnight with rabbit anti-NLRP3 (1:250; Abcam, Cambridge, UK) and rabbit anti-STAT1 (1:500; Abcam, Cambridge, UK) antibodies following routine deparaffinization and hydration. The sections were then incubated with a secondary antibody (goat anti-rabbit IgG-HRP) and stained with hematoxylin. Image-Pro Plus software (Media Cybernetics) was used to analyze the percentage area of immunopositive staining and the average optical density in both valvular leaflets and interleaflet triangles. The average optical density values were computed using the following formula: AOD (average optical density) = IOD (integrated optical density)/area.

### Immunofluorescence

The paraffin sections were dewaxed and subjected to Tris-EDTA antigen retrieval (pH 8.0). Valve interstitial cells (VICs) cultured in vitro were fixed in 4% paraformaldehyde. The cells were then treated with 0.1% Triton X-100 for 15 min, followed by epitope blocking for one hour in 5% BSA in PBS. Primary antibody staining was conducted overnight at 4 °C in a blocking solution containing mouse anti-CD4 (1:200; Abcam, Cambridge, UK), rabbit anti-IFN-γ (1:100; Abcam, Cambridge, UK), rabbit anti-STAT1 (1:200; Abcam, Cambridge, UK), rabbit anti-NLRP3 (1:200; Abcam, Cambridge, UK), mouse anti-caspase-1 (1:200; Abcam, Cambridge, UK), rabbit anti-RUNX2 (1:200; Abcam, Cambridge, UK), rabbit anti-BMP2 (1:200; Abcam, Cambridge, UK), or rabbit anti-α-SMA (1:200; Abcam, Cambridge, UK). The sections were then stained with goat anti-rabbit IgG-AF488, donkey anti-mouse IgG-AF555 or goat anti-rabbit IgG-AF750 (1:1000; Abcam, Cambridge, UK) secondary antibodies for 1 h at room temperature, followed by DAPI staining (Solaibao, Beijing, China). The sections were imaged at magnifications ranging from 4× to 60×. CD4^+^ and IFN-γ^+^ cells were counted manually and normalized to the area of the DAPI mask corresponding to the leaflet. The immunofluorescence intensities of NLRP3, Caspase-1, RUNX2, BMP2 and α-SMA were calculated by ImageJ.

### Flow cytometry

Splenic single-cell suspensions were extracted following established protocols. Macrophages cultured in vitro were digested into single-cell suspensions by Accutase (Invitrogen, Carlsbad, USA). T cells were stimulated with PMA (50 ng/mL; Cat. HY-18,739; MCE, USA) and ionomycin (1 µg/mL; Cat. HY-13,434; MCE, USA) for 6 h. BFA (10 µg/mL; Cat. HY-16,592; MCE, USA) was introduced during the last 4 h to induce cytokine secretion. To prevent nonspecific staining, single-cell suspensions were preincubated with anti-CD16/CD32 antibodies (clone 2.4G2; Fc block; BD Biosciences) for 15 min. Then, the cells were stained with anti-CD4 (eBioscience, Carlsbad, USA), anti-CD45 (eBioscience, Carlsbad, USA), anti-F4/80 (eBioscience, Carlsbad, USA), anti-CD11b (eBioscience, Carlsbad, USA), anti-Ly6C (eBioscience, Carlsbad, USA), or anti-Ly6G (eBioscience, Carlsbad, USA) antibodies. For intracellular staining, cells were treated with anti-IFN-γ, anti-NLRP3 (R&D Systems, Minnesota, USA), or anti-caspase-1 (Genetex, San Antonio, USA) antibodies, followed by fixation and permeabilization using the Intracellular Fixation & Permeabilization Buffer Set (eBioscience, USA). Macrophages were identified as CD45^+^CD11b^+^F4/80^+^, and monocytes were identified as CD45^+^CD11b^+^Ly6G^-^. The spleen and heart samples were analyzed using a FACSVerse Flow Cytometer and a FACSCanto Flow Cytometer (BD Biosciences). FlowJo V10 software (BD Biosciences) was used for data analysis.

### Th1 induction

Spleens from WT C57BL/6J mice were gently crushed, and red blood cells were lysed in erythrocyte lysis buffer (Solarbio, China) and washed thoroughly with PBS. The resulting splenocyte suspensions were filtered through a 70-µm sieve (BD Biosciences, USA). Naive CD4^+^ T cells were isolated using a mouse naive CD4^+^ T-cell isolation kit (Cat. 130-104-453; Miltenyi Biotec, Germany) according to the manufacturer’s instructions. The isolated naive T cells were induced to differentiate into Th1 cells by adding 20 ng/ml IL-2 (Proteintech, Chicago, USA) and 10 µg/ml anti-IL-4 (BioXcell, NH, USA).

### Coculture of BMDMs and Th1 cells

BMDMs were extracted from WT C57BL/6J mice as previously described and induced with 20 ng/ml M-CSF for 7 days (Dorighello et al. 2022). Subsequently, the BMDMs were stimulated with ox-LDL for 48 h, and the ATP intervention group was pretreated with 3 mM ATP for 30 min. For the CY-09 intervention group, BMDMs were treated with 5 µM CY-09 for 48 h. In vitro coculture assays were conducted using cell culture inserts featuring porous polycarbonate filters with a pore size of 0.4 μm placed within 24-well plates. Th1 cells (5 × 10^6^) were added to the upper chamber of the Transwell chamber, and BMDMs were treated with ox-LDL and ATP in the lower chamber for 2.5 days. Then, BMDMs were collected for testing or cultured with fresh medium for 2 days to obtain conditioned medium for subsequent experiments with VICs.

### VICs isolation and culture

Murine VICs were isolated from noncalcified aortic valves of WT C57BL/6J mice via collagenase digestion as previously reported (Zeng et al. 2017). These interstitial cells were cultured in M199 complete medium at 37 °C under 5% CO2. Cells derived from passages 3 to 5 were chosen for experiments. To induce calcification, VICs at a concentration of 5 × 10^5^ cells/mL were cultured in M199 medium supplemented with 5% fetal bovine serum, 50 µg/mL ascorbic acid, 100 nM dexamethasone, and 10 mM β-glycerophosphoric acid as previously described(Li et al. 2017). To examine the impact of macrophages on the osteogenic differentiation of VICs, 1 mL of nonconditioned (fresh) medium or conditioned medium obtained from macrophages after intervention as previously described was added to the VICs procalcifying medium. After a 7-day incubation period, the harvested cells were prepared for experiments. To characterize osteoblast-like phenotypes, ALP and alizarin red staining were performed using an ALP detection kit and alizarin red S staining solution (Biyuntian, Shanghai, China), and the OD at 405 nm was used to determine ALP activity according to the manufacturer’s instructions.

### ELISA

The cell culture supernatant was centrifuged at 300 × g for 5 min, aliquoted and stored at − 80 °C. The murine cytokines IL-1β and IL-18 were detected by enzyme-linked immunosorbent assay (ELISA) according to the manufacturer’s instructions (Wuhan Huamei Bioengineering Co., Ltd., China).

## Results

### CAVS progression is correlated with the activation of the IFN-γ-driven Th1-type response

**Fig. 1:**
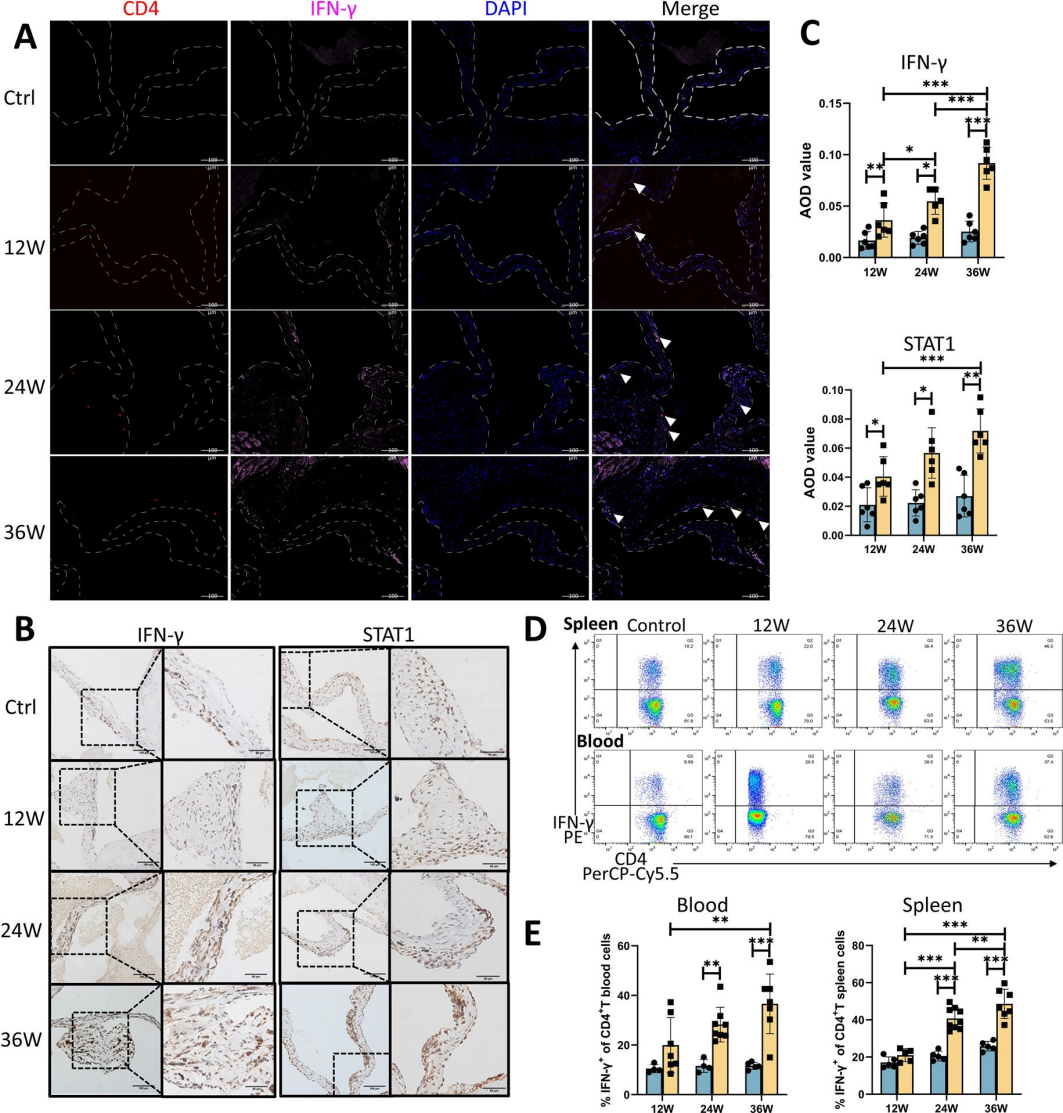
CAVS mice exhibit valvular and peripheral Th1 expansion, as well as IFN-γ signaling activation. (**A**) Representative immunofluorescence. Red: CD4; pink: IFN-γ (original magnification, 200×; Scale bars = 100 μm). (**B**) Representative immunohistochemical images of IFN-γ and STAT1 in aortic valves from different time points (Left, original magnification, 200×; Scale bars = 100 μm; Right, original magnification, 400×; Scale bars = 50 μm). (**C**) The statistics of the average optical density (AOD) values of IFN-γ and STAT1 are shown (*n* = 6/group). Representative flow cytometry plots (**D**) and the statistical results (**E**) of IFN-γ effects on CD4^+^ T cells in the blood and spleen of CAVS mice at 12, 24, and 36 weeks (*n* = 5–7/group). Q2-UR represents CD4^+^IFN-γ^+^ T cells. The control group in A, B, and D were derived from mice at week 24. Data are presented as the means ± SEMs. *p < 0.05; **p < 0.01; ***p < 0.001; *, **, *** indicate differences between groups. Calcific aortic valve stenosis, CAVS. T helper 1, Th1. Interferon-gamma, IFN-γ. AOD, average optical density

Researchers have reported pronounced leaflet thickening and valve calcification, accompanied by a significant decrease in aortic valve systolic dimension and an increase in peak flow velocity, after HFD for 24 to 36 weeks (Jung et al. 2015). After 12 weeks of HFD, valve function was normal, and no calcification was observed. However, the number of IFN-γ-expressing Th1 cells and the protein expression levels of IFN-γ and STAT1 in the aortic valve were greater in the CAVS group than in the control group, and these levels gradually increased as CAVS progressed (Fig. 1A -C). Similar to the effects observed in the aortic valve, the proportions of Th1 cells in the spleen and peripheral blood were significantly greater of CAVS mice than control mice at 24 and 36 weeks (Fig. 1D and E). These results suggest that Th1 cells and IFN-γ signaling are involved in the early immune activation of CAVS.

### IFN-γ aggravates valve calcification and dysfunction in ApoE−/− mice

**Fig. 2:**
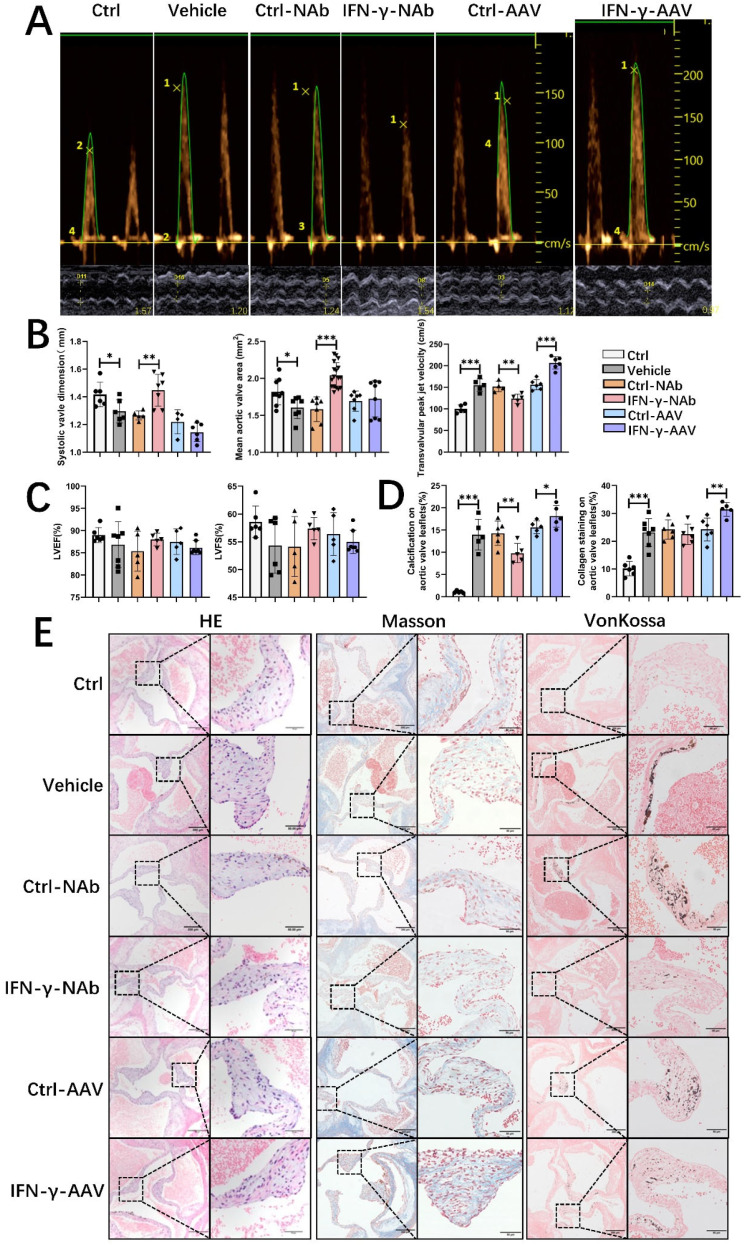
IFN-γ aggravates valve calcification and dysfunction. (**A**) Representative plots of the transvalvular peak jet velocity and systolic valve dimensions of different groups of mice evaluated using continuous-wave Doppler. (**B**) Quantitative analysis of the mean aortic valve area, transvalvular peak jet velocity, and systolic valve dimensions as assessed by echocardiography (*n* = 5–15/group). (**C**) Quantitative analysis of LVEF and LVFS in different groups of mice (*n* = 5–7/group). (**D**) Statistical analysis of calcification and collagen staining of aortic valve leaflets (*n* = 6/group). (**E**) Representative images of HE, Von Kossa, and Masson staining of aortic valve leaflets and interleaflet triangle sections showing calcified and fibrotic areas (left, original magnification, 100×; scale bars = 200 μm; right, original magnification, 400×; scale bars = 50 μm). The data are presented as means ± SEMs. *p < 0.05; **p < 0.01; ***p < 0.001; *, **, *** indicate significant differences between groups. Interferon-gamma, IFN-γ. Left ventricular ejection fraction, LVEF. Left ventricular fraction shortening, LVFS

To determine the role of IFN-γ, we used IFN-γ-overexpressing adenovirus type 9 (AAV9) and anti-IFN-γ neutralizing antibodies. Compared with other serotypes, AAV9 offers more effective cardiac gene delivery(Bish et al. 2008). Compared with control antibody treatment, IFN-γ blockade significantly increased the leaflet separation and mean valve area and decreased the transvalvular peak jet velocity (Fig. 2A and B). Overexpression of IFN-γ resulted in a significant increase in transvalvular flow velocity (Fig. 2A and B). Neither anti-IFN-γ antibodies nor IFN-γ AAV9 affected LVEF or LVFS (Fig. 2C). Body weight and blood lipid and glucose levels are shown in Supplementary Table S1. The histological analysis of aortic valve tissue in CAVS mice indicated a progressive thickening of the leaflets and an increase in collagen accumulation and valve calcification over time (Fig. 2E). The percentages of calcification and collagen area in the aortic valve were significantly greater in IFN-γ AAV9-treated mice than in control-AAV9 mice (Fig. 2D). Blocking IFN-γ significantly reduced the degree of valve calcification, as shown by von Kossa staining (Fig. 2D). In brief, IFN-γ aggravates aortic valve calcification and stenosis to promote the progression of CAVS.

### IFN-γ downregulates NLRP3 activation in Ly6C^+^ monocytes and macrophages in CAVS mice

**Fig. 3:**
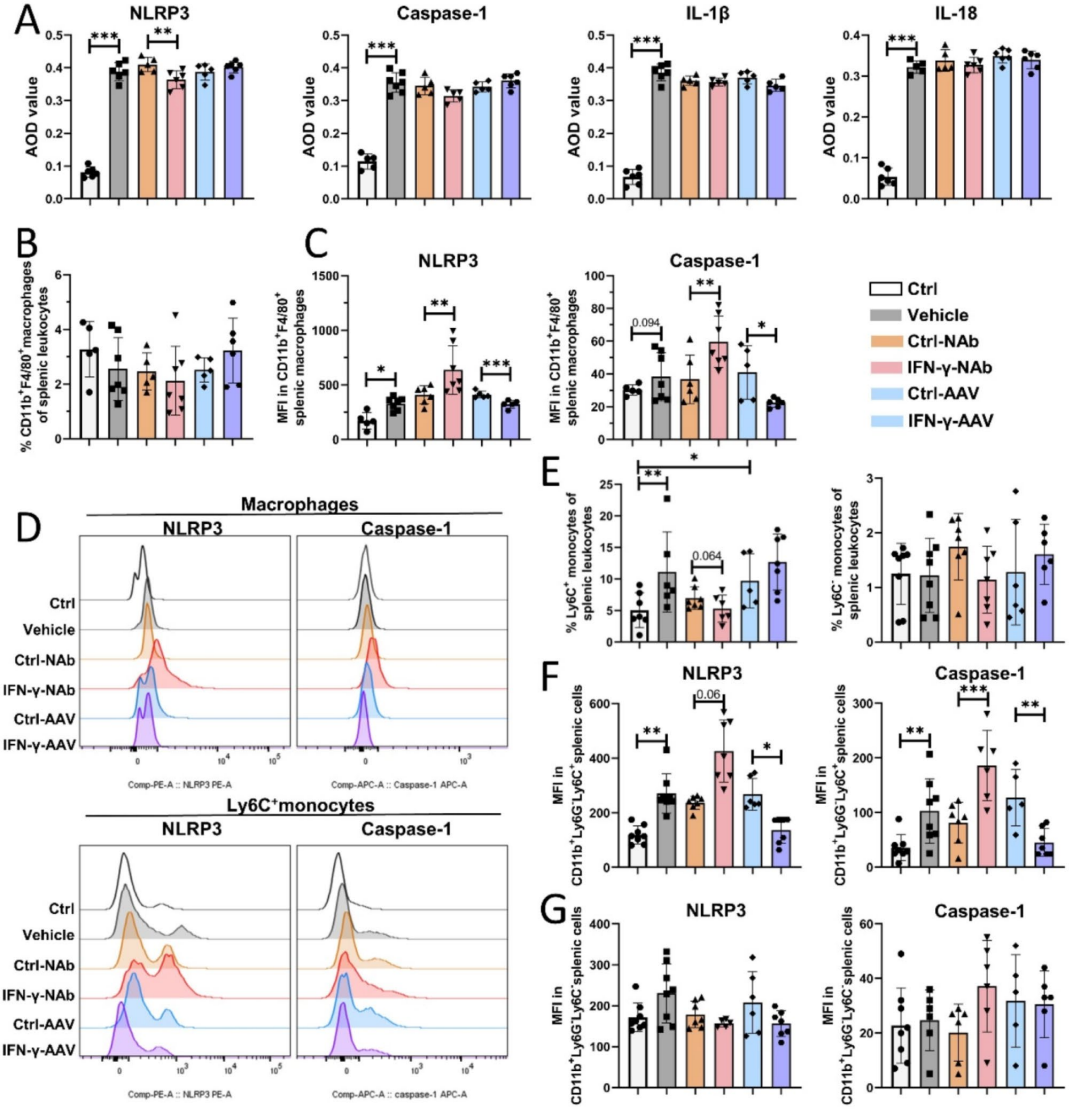
IFN-γ inhibits the activation of the NLRP3 inflammasome in macrophages and Ly6C^+^ monocytes from CAVS mice. (**A**) The average optical density (AOD) values of duplicate values of NLRP3, caspase-1, IL-1β, and IL-18 in the aortic valve. (**B**) The statistical results of the frequencies of CD11b^+^F4/80^+^ macrophages in total splenic leukocytes (*n* = 5–7/group). (**C**) Statistical analysis of the mean fluorescence intensity (MFI) of NLRP3 and caspase-1 in splenic macrophages (*n* = 5–7/group). (**D**) Line graph of the MFI of NLRP3 and caspase-1 expression in splenic macrophages and Ly6C^+^ monocytes in the different groups. (**E**) The statistical results of the frequencies of CD11b^+^Ly6G-Ly6C^+^ and CD11b^+^Ly6G-Ly6C- monocytes in total splenic leukocytes (*n* = 5–7/group). The statistical results of the MFI of NLRP3 and caspase-1 expression in splenic CD11b^+^Ly6G-Ly6C^+^ (F) and CD11b^+^Ly6G-Ly6C- (G) monocytes (*n* = 6–8/group). The data are presented as means ± SEMs. *p < 0.05; **p < 0.01; ***p < 0.001; *, **, *** indicate significant differences between groups. Interferon-gamma, IFN-γ. Average optical density, AOD. Mean fluorescence intensity, MFI

The vascular inflammatory response is closely related to endothelial damage, plaque formation, lipid metabolism, and smooth muscle cell function. IFN-γ has both proinflammatory and anti-inflammatory properties and thus exerts diverse effects through different mechanisms in vascular diseases (Niwa et al. 2004; King et al. 2009; Christen et al. 1994). We previously identified NLRP3 signaling activation in innate immunity as a pivotal inflammatory target for preventing CAVS progression. Here, we further explored whether intervention to suppress or overexpress IFN-γ affects the NLRP3 pathway. Compared with control treatment, anti-IFN-γ antibody treatment reduced NLRP3 expression in the aortic valve, and there were no significant differences in downstream caspase-1, IL-1β, or IL-18 levels, which could be attributed to nonspecific total cellular protein expression (Fig. 3A). Therefore, we further examined splenic macrophages and monocytes and determined that the frequency of splenic macrophages to total splenic leukocytes in CAVS mice was not significantly different from that in control mice, whereas the frequency of splenic Ly6C^+^ monocytes was elevated (Fig. 3B, D and E). However, IFN-γ blockade significantly elevated the expression of NLRP3 and caspase-1 in macrophages, whereas IFN-γ overexpression had the opposite effect (Fig. 3C). This phenomenon was also observed in splenic Ly6C^+^ monocytes, but the number of Ly6C^-^ monocytes was not affected by IFN-γ intervention (Fig. 3D, F and G). Collectively, these data show that IFN-γ interacts with NLRP3 to inhibit the activation of the NLRP3 inflammasome.

### Th1 cells inhibit NLPR3 in ox-LDL-treated macrophages through the IFN-γR1/IFN-γR2-STAT1 pathway

**Fig. 4:**
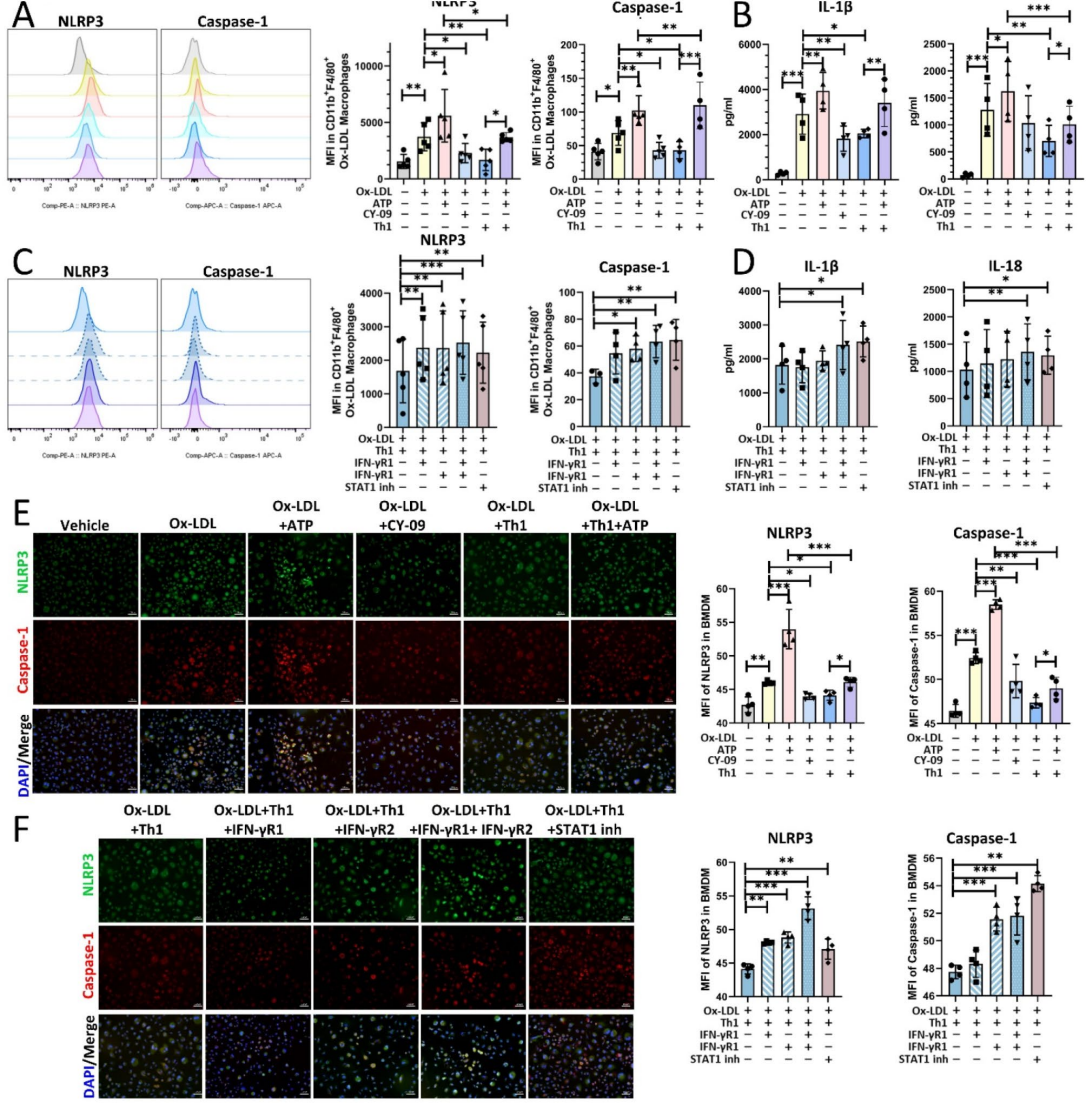
Th1 cells suppress the NLRP3 inflammasome via the IFN-γR1/IFN-γR2-STAT1 pathway. (**A** and **C**) Left, representative histogram of the MFI of NLRP3 and caspase-1 in different groups of BMDMs as determined by flow cytometry. Right, the statistical results of the MFI of NLRP3 and caspase-1 (*n* = 4/group). (**B** and **D**) Statistical data of IL-1β and IL-18 concentrations in the cell supernatant of each group (*n* = 4/group). (**E** and **F**) Left, representative images of immunofluorescence images of BMDMs in each group. Right, the MFI of NLRP3 and caspase-1 as determined by immunofluorescence in different groups of BMDMs (*n* = 4/group) (original magnification, 100×; scale bars = 100 μm). Representative immunofluorescence images of the Ox-LAL + Th1 group are shown and are consistent with the groups in the bar graph on the right. The data are presented as means ± SEMs. *p < 0.05; **p < 0.01; ***p < 0.001; *, **, *** indicate significant differences between groups. Interferon-gamma, IFN-γ. Mean fluorescence intensity, MFI

To further explore the mechanism by which IFN-γ blocks the NLRP3 inflammasome in BMDMs, we induced the differentiation of sorted naive T cells into CD4^+^ Th1 cells, which were then cocultured with ox-LDL-treated macrophages in vitro. The addition of ox-LDL activated the NLRP3 pathway in BMDMs, and the NLRP3 agonist ATP or the NLRP3 inhibitor CY-09 significantly upregulated or downregulated NLRP3 activation, respectively (Fig. 4A and B, and 4E). Coculture with Th1 cells significantly reduced the protein expression level of NLRP3 in BMDMs. When ATP was added to the coculture system, the activation of the NLRP3 signaling pathway in BMDMs was significantly upregulated but was still lower than that in the ATP-only group without Th1 cells (Fig. 4A and B, and 4E). The addition of neutralizing antibodies against IFN-γR1 and IFN-γR2 and pretreatment with a STAT1 inhibitor significantly weakened the inhibitory effect of Th1 cells on NLRP3 activation in BMDMs, indicating that Th1 cells inhibit NLRP3 activation in ox-LDL-treated macrophages through the IFN-γR1/IFN-γR2-STAT1 pathway (Fig. 4C and D, and 4F).

### Inhibition of NLRP3 activation by Th1 cells reduces the osteogenic calcification of valvular interstitial cells induced by conditioned medium from BMDMs

**Fig. 5:**
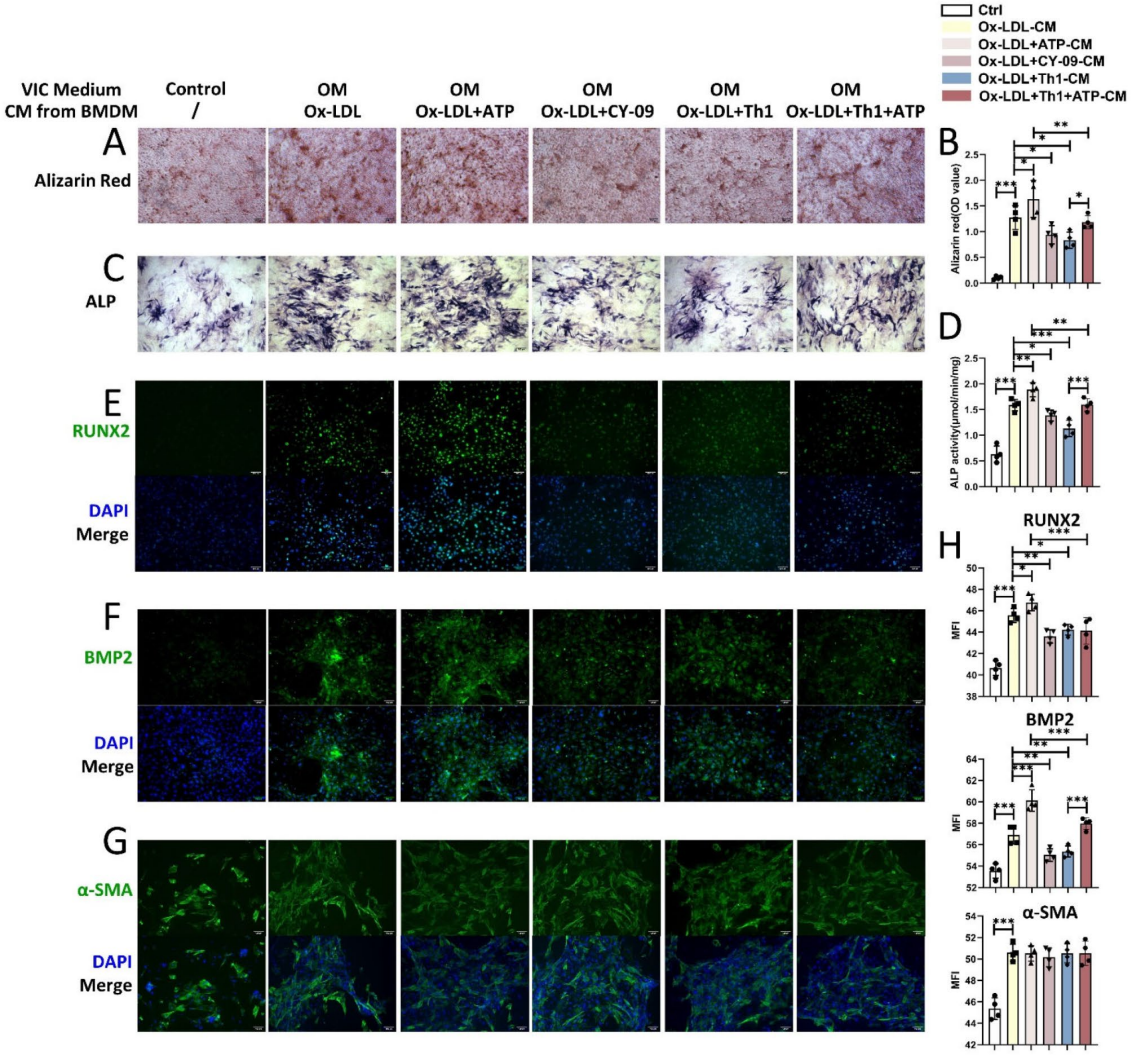
Th1 cells inhibit the enhancement of VICs calcification by conditioned medium from NLRP3-activated macrophages. Representative images of alizarin red staining (**A**) and ALP staining (**C**) of VICs from different groups (original magnification, 50×; scale bars = 100 μm). (**B**) Statistical analysis of optical density (OD) values of VICs after alizarin red staining (*n* = 4/group). (**D**) Statistical analysis of ALP activity in VICs (*n* = 4/group). Representative images of VICs immunofluorescence in each group. Green: RUNX2 (E), BMP2 (**F**), and α-SMA (**G**) (original magnification, 100×; scale bars = 100 μm). (D) Statistical analysis of the MFI of RUNX2, BMP2, and α-SMA immunofluorescence in different groups of VICs (*n* = 4/group). The data are presented as means ± SEMs. *p < 0.05; **p < 0.01; ***p < 0.001; *, **, *** indicate significant differences between groups. Mean fluorescence intensity, MFI. Alkaline phosphatase, ALP. Runt-related transcription factor 2, RUNX2. Bone morphogenetic protein, BMP. Alpha-smooth muscle actin, α-SMA

Conditioned medium containing IL-6 and TNF-α secreted by M1 macrophages can significantly promote the osteogenic calcification of VICs^6^. We found that conditioned medium from ATP-treated BMDMs further enhanced the pro-osteogenic effect of ox-LDL and significantly increased alizarin red staining and ALP, RUNX2 and BMP2 protein levels, whereas CY-09 addition had the opposite effects (Fig. 5A- F, and 5H). This finding suggests that the activation of the NLRP3 signaling pathway in macrophages promotes VICs osteoblastic calcification through soluble factors. Since we observed that Th1 and IFN-γ significantly reduced the expression of the NLRP3 pathway in macrophages, we further treated VICs with fresh conditioned medium after coculture. Coculture with Th1 cells significantly reduced the pro-osteogenic effect of the conditioned medium of macrophages on VICs calcification (Fig. 5A-F, and 5H). Although ATP weakened the inhibitory effect of Th1 cells, the pro-osteogenic effect of the conditioned medium was still significantly lower than that in the ATP-only group (Fig. 5A-F, and 5H). The addition of osteogenic medium upregulated the protein expression of α-SMA in VICs, but α-SMA expression did not differ significantly between groups treated with different conditioned media, indicating that NLRP3 signaling in macrophages did not affect myofibroblast calcification (Fig. 5G and H). These findings show that Th1 cells play a protective role by inhibiting the expression of the NLRP3 signaling pathway in macrophages to reduce osteoblastic calcification in VICs.

## Discussion

Th1 cell infiltration increased significantly in the aortic valves of CAVS mice, accompanied by an increase in circulating Th1 cells. IFN-γ impaired aortic valve function and promoted valve calcification. Th1 cells downregulate the NLRP3 signaling pathway in macrophages through the IFN-γR1/IFN-γR2-stat1 pathway, which can reduce the osteoblastic calcification of valve interstitial cells.

Cardiac macrophages can be divided into tissue-resident CCR2^−^ macrophages (mainly derived from the embryonic yolk sac period) and CCR2^+^ macrophages (mainly derived from circulating monocytes) (Epelman et al. 2014). The majority of macrophages in the valves of healthy mice are CX3CR1^high^/Ly6C^−^/CCR2^−^ (Raddatz et al. 2020). Classical Ly6C^+^ monocytes enter the heart through CCL2-CCR2 signaling and transform into CCR2^+^ macrophages in an inflammatory environment (Wynn et al. 2013). Nonclassical Ly6C^-^ monocytes are widely considered anti-inflammatory and maintain vascular homeostasis by “patrolling” the vasculature in a LFA-1 integrin-dependent manner (Auffray et al. 2007; Carlin et al. 2013). We detected peripheral Ly6C^+^ monocytosis in CAVS mice but no significant changes in Ly6C^−^ monocytes (Fig. 3). Mouse Ly6C^+^ and Ly6C^−^ monocytes are comparable to CD14^+^ monocytes(CD16^−^classical and CD16^+^intermediate subsets) and non-classical CD14^−^CD16^+^ subsets in human monocytes (Ingersoll et al. 2010; Schmidl et al. 2014). Clinical studies have reported that the valve function of patients with severe CAVS is associated with circulating intermediate monocyte levels, which can be reduced after percutaneous aortic valve replacement (Hewing et al. 2017a, b). However, the effect of blocking the CCL2-CCR2 axis in vivo on Ly6C^+^ monocyte mobilization and CAVS progression remains unexplored. Researchers have only observed in vitro that silencing CCR2 can inhibit the osteogenic differentiation of VICs, whereas recombinant CCL2 protein shows the opposite effect (Zhu et al. 2019).

We observed that foam cells facilitated a phenotypic shift in VICs in vitro (Fig. 4). Following lipid infiltration and foam cell formation, osteoblast-like cells appear within the aortic valve leaflet and initiate calcification (Youssef et al. 2021). CAVS and atherosclerosis have similar risk factors and downstream mediators; hyperlipidemia can trigger valve calcification and hemodynamic changes in mice (Aikawa et al. 2007; Weiss et al. 2006). However, recent studies have shown that a high-fat diet can induce trained immunity, thereby impacting the innate immune response. This results in a significant increase in the absolute number, activation state, and secretion of inflammatory cytokines and chemokines by myeloid cells in circulation. Additionally, NLRP3 inflammasome is involved in transcriptomic and epigenomic reprogramming of myeloid progenitor cells, leading to increased proliferation and an enhanced innate immune response (Christ et al. 2018). In this study, we found significant activation of the NLRP3 pathway in splenic macrophages and Ly6C^+^ monocytes in CAVS mice (Fig. 3). In the heart, CCR2^+^ macrophages are enriched in genes that regulate the NLRP3 inflammasome, and the production of IL-1β under in vivo cardiac stress mainly depends on the expansion of CCR2^+^ monocytes/macrophages (Epelman et al. 2014). In addition, NLRP3-dependent IL-1β levels are elevated in circulating monocytes, and there is evidence of systemic inflammation in patients with severe CAVS(Abplanalp et al. 2020). Therefore, the activation and dysregulation of the NLRP3 inflammasome in innate immune cells are closely related to the immunopathological mechanism of CAVS.

The expression of IFN-γ increases with chronic inflammation in atherosclerosis (Dinh et al. 2017), and a lack of IFN-γ and T-bet can significantly reduce the percentages of lesional macrophages and smooth muscle content (Buono et al. 2005). Although IFN-γ deficiency does not affect serum cholesterol levels or lipoprotein profiles, it exacerbates the degree of atherosclerosis (Buono et al. 2003). The activated immune cells in the diseased aortic valve secrete inflammatory mediators that promote the formation of calcified nodules. IFN-γ potently promotes angiogenesis, inflammation and osteogenesis and can induce the osteogenic phenotype of VICs through the JAK/STAT and ERK/HIF-1α pathways in vitro(Parra-Izquierdo et al. 2019). Our experiments with IFN-γ intervention in vivo further corroborated these results in vitro; IFN-γ could significantly aggravate aortic valve stenosis and dysfunction (Fig. 2). IFN-γ is mainly secreted by activated T lymphocytes and natural killer cells (Burke and Young 2019). Infiltration of the aortic valve by the CD4^+^ T-cell subset predominated over infiltration by the CD8^+^ subset (Steiner et al. 2012). IFN-γ secreted by CD8^+^ T cells can promote the production of bone marrow mononuclear cells and increase the level of circulating monocytes in mice with hypercholesterolemia, thereby promoting atherosclerosis (Cochain et al. 2015). In addition, CD8^+^ cell-derived IFN-γ reduces the number of osteoclasts and inhibits their calcium absorption function, leading to an increase in the calcium content of the aortic valve (Nagy et al. 2017). However, there are few studies of the immune mechanism of Th1 cells in CAVS. In general, IFN-γ secreted by CD4^+^ T cells promotes the transformation of macrophages to proinflammatory M1 macrophages, enhancing their ability to engulf and digest pathogens. In addition, IFN-γ activates inducible nitric oxide synthase (NOS2 or iNOS) to produce NO, which kills bacteria in cells (Belardelli 1995). Surprisingly, we have found that the in vitro study of IFN-γ intervention in macrophages contradicts the exacerbation of CAVS by IFN-γ observed in vivo and the anticipated outcomes from previous research. The inflammatory mediators secreted by macrophages after IFN-γ intervention can inhibit osteogenic differentiation of VICs, and NLRP3 of macrophages may serve an important negative regulatory target for IFN-γ (Figs. 3 and 5).Although the expression of NLRP3 increases significantly after macrophage polarization to the M1 phenotype, NLRP3 expression is mainly induced by lipopolysaccharide rather than IFN-γ (Awad et al. 2017). We found that IFN-γ inhibited the NLRP3 inflammasome on macrophages, thereby reducing the secretion of IL-1β and IL-18. In turn, the proinflammatory cytokine IL-18 can induce and amplify the Th1 immune response (Sica and Mantovani 2012), which may be considered a negative feedback mechanism between Th1 cells and macrophages that limits the severity of inflammation.

IFN-γ deficiency leads to arthritis after Brucella infection. IFN-γ secreted by lymphocytes, such as innate lymphocytes (ILCs), can induce iNOS. Nitric oxide directly inhibits the assembly and activation of the NLRP3-caspase-1 inflammasome in Brucella-infected macrophages via thiol nitrosylation, which reduces the production of IL-1β (Mishra et al. 2013; Lacey et al. 2019). IFN-γ also has a protective effect on the progression of T1D diabetes (Lee et al. 2008); however, macrophages from diabetic mice have developmental and immune dysfunction and are resistant to apoptosis due to their low responsiveness to IFN-γ after activation (Lee et al. 2012). IFN-γR1, which is constitutively expressed on all nucleated cells, is responsible for binding to IFN-γ. IFN-γR2 subunits are generally thought to be the limiting factor for responsiveness to IFN-γ, and their expression is regulated by cell type, differentiation or activation state (Xu et al. 2018). The IFN-γ receptor further triggers intracellular signaling through the signal transducer and activator of transcription (STAT) pathway (Schoenborn and Wilson 2007). The hyporesponsiveness of NOD macrophages to IFN-γ is associated with low expression of interferon-γ receptor 2 (IFN-γR2) and abnormal regulation of downstream JAK-STAT signal transduction (Lee et al. 2012). We found that IFN-γ inhibited the activation of macrophages via the NLRP3 inflammasome through IFN-γR1/IFN-γR2 and the downstream STAT1 signaling pathway. Other studies have shown that the expression of IFN-γR1 is significantly upregulated in calcified valve tissues. In addition, the protein expression of IFN-γR and STAT1 in calcified valve tissues is greater in men than in women, consistent with sex differences reported in comparative clinical studies (Aggarwal et al. 2013). Therefore, IFN-γR1, IFN-γR2 and STAT signaling are involved in the ability of IFN-γ to limit NLRP3-induced inflammation. The macrophages in diseased valves may have specific defects in the negative regulatory response to Th1 cells, resulting in local tissue damage and CAVS pathogenesis.

This study elucidates the negative regulatory role of IFN-γ in CAVS. IFN-γ inhibits macrophage NLRP3 activation, thereby limiting inflammation and reducing VICs calcification. Our findings contribute a better understanding of the anti-inflammatory mechanisms of innate immune cells in CAVS and will support translational studies of cellular therapies for precision medicine.

### Electronic supplementary material

Below is the link to the electronic supplementary material.

Supplementary Material 1

## Data Availability

The datasets used and analyzed during the current study are available from the corresponding author upon reasonable request.

## Abbreviations

CAVS: Calcific Aortic Valve Stenosis
VICs: Valve Interstitial Cells
NLRP3: The NLR family Pyrin Domain Containing 3
PAMPs: Pathogen-Associated Molecular Patterns
DAMPs: Damage-Associated Molecular Patterns
ASC: Apoptosis-Associated Speck-like protein Containing a CARD
IFN-γ: Interferon-γ
Th1: Helper type 1 T cells
JAK/STAT: Anus Kinase/Signal Transducer and Activator of Transcription
NO: Nitric oxide
BMDMs: Bone Marrow–Derived Macrophages
HFD: High-Fat Diet
AAV9: Adeno-Associated Virus 9
