# Supplementary material for: Th1 cells reduce the osteoblast-like phenotype in valvular interstitial cells by inhibiting NLRP3 inflammasome activation in macrophages
Source: Mol Med. 2024 Jul 30;30:110. doi: 10.1186/s10020-024-00882-z (PMC11287975; doi:10.1186/s10020-024-00882-z)
Supplement: Supplementary file 1 — Supplementary Material 1 [file 10020_2024_882_MOESM1_ESM.docx]

**Supplementary Table 1**. Comparison of the Animal Characteristics and Metabolic Parameters of ApoE−/− Mice in Different Groups

|  | Ctrl | Vehicle | Ctrl-NAb | IFN-γ-NAb | Ctrl-AAV | IFN-γ-AAV |
| --- | --- | --- | --- | --- | --- | --- |
| BW before（g） | 21.41 ±2.35 | 23.55 ±1.89 | 22.86 ±2.12 | 23.45 ±2.74 | 21.6 ±1.88 | 22.75 ±2.06 |
| BW after （g） | 29.51 ±2.11 | 46.56 ±1.69 | 43.63 ±2.66 | 46.85 ±2.48 | 45.88 ±2.36 | 47.57±2.24 |
| Glucose (mmol/L) | 9.85± 1.65 | 10.12± 1.36 | 10.25± 2.47 | 9.89±2.32 | 10.42±1.96 | 9.78±1.78 |
| TC (mmol/L) | 12.76± 1.84 | 25.66±3.51 | 28.62± 3.75 | 27.08±5.00 | 26.36±4.00 | 27.45±4.21 |
| LDL (mmol/L) | 8.26± 1.22 | 27.54±4.52 | 26.85± 3.96 | 28.66±5.65 | 26.95±3.15 | 27.48±4.61 |
| TG (mmol/L) | 1.10± 0.32 | 2.21±0.25 | 2.05± 0.62 | 2.34± 0.47 | 2.15±0.15 | 2.37±0.62 |

The data are presented as the means ± SEMs. **p*<0.05, ***p* < 0.01 vs. control group. #*p*<0.05, ##*p* < 0.01 vs. CAVS group. N=8. AVA, aortic valve area. AVAi, AVA indexed for body weight. Max Vel, maximal transvalvular velocities by continuous-wave Doppler. LVEDD, left ventricular end-diastolic internal diameter. LVESD, left ventricular end-systolic internal diameter. LVEF, left ventricular ejection fraction. LVFS, left ventricular fractional shortening.
